# Dataset on the piezo-spectroscopic behaviour of hydroxylapatite: Effect of mechanical stress on the Raman and Infrared vibrational bands from *ab initio* quantum mechanical simulations

**DOI:** 10.1016/j.dib.2018.03.035

**Published:** 2018-03-13

**Authors:** Gianfranco Ulian, Giovanni Valdrè

**Affiliations:** Dipartimento di Scienze Biologiche, Geologiche e Ambientali, Centro di Ricerche Interdisciplinari di Biomineralogia, Cristallografia e Biomateriali, Università di Bologna “Alma Mater Studiorum”, Piazza di Porta San Donato 1, 40126 Bologna, Italy

## Abstract

This article reports data on the vibrational (Raman and Infrared) behavior of hydroxylapatite [OHAp, Ca_10_(PO_4_)_6_(OH)_2_, space group *P*6_3_] under mechanical stress, which were discussed in details in the work of Ulian and Valdrè (2017) [1]. The dataset has been obtained by *ab initio* quantum mechanical means, by employing Density Functional Theory methods, in particular the B3LYP hybrid functional, all-electron Gaussian-type orbitals basis sets and a correction to take into account the effects of dispersive forces.

## Specifications Table

**Table:** 

Subject area	*Physics*
More specific subject area	*Vibrational spectroscopy (Raman and IR) of biomaterials*
Type of data	*Tables*
How data was acquired	*Quantum mechanical simulations at the Density Functional Theory (DFT)/B3LYP level of theory, including dispersive forces contributions (CRYSTAL14 code)*
Data format	*Raw, analyzed*
Theoretical factors	*Starting geometry taken from previous DFT simulations*[2].
Theoretical features	*Quantum mechanical simulations conducted using Density Functional Theory, B3LYP functional and Gaussian-type orbitals basis sets.*
	Inclusion of dispersive forces contribution via DFT-D2 scheme, corrected for the B3LYP functional (B3LYP-D* approach).
	*Geometry optimization of the unit cell with and without applied strains.*
Data source location	*Bologna, P. Porta San Donato 1, Italy*
Data accessibility	*Data is displayed within this article.*
Related research article	*This Data in Brief article is submitted as a companion paper to: Ulian, G. & Valdrè, G. (2017) Effect of mechanical stress on the Raman and Infrared bands of hydroxylapatite: a quantum mechanical first principle investigation. Journal of the Mechanical Behavior of Biomedical Materials, in press.*

## Value of the data

• Geometries of hydroxylapatite [OHAp, Ca_10_(PO_4_)_6_(OH)_2_, space group *P*6_3_] at both equilibrium and stressed conditions.
• Vibrational analysis (Raman and IR) of each hydroxylapatite geometry.
• Vibrational spectra (Raman and IR) of each hydroxylapatite geometry up to 4000 cm^−1^, which could be employed for comparison with experimental data.
• Results obtained at the Density Functional Theory (DFT) level, employing hybrid B3LYP functional and including a correction to take into account the contribution of dispersive forces.

## 1 Data

### 1.1 Hydroxylapatite geometry at equilibrium and under mechanical stress

Equilibrium and deformed (strained) OHAp models were realized and geometrically optimized, and the stress for each deformation was calculated according to stress-strain formulations.

Hydroxylapatite (OHAp, s.g. *P*6_3_) was optimized to take into account the effect of dispersive force contribution in the final unit cell and internal geometries (Table 1). Then, it was deformed according to the three symmetry-independent strains related to the *P*6_3_ space group [1]:

(1) $$\varepsilon_{1}=\delta \left(\begin{array}{ccc} 1 & 0 & 0\\ 0 & 0 & 0\\ 0 & 0 & 0 \end{array}\right)\;\varepsilon_{3}=\delta \left(\begin{array}{ccc} 0 & 0 & 0\\ 0 & 0 & 0\\ 0 & 0 & 1 \end{array}\right)\;\varepsilon_{4}=\delta \left(\begin{array}{ccc} 0 & 0 & 0\\ 0 & 0 & 1\\ 0 & 1 & 0 \end{array}\right)$$

where *ε*_1_ and *ε*_3_ are normal strains (uniaxial) perpendicular to the (100) and (001) surfaces of the hexagonal unit cell and *ε*_4_ is a shear stress parallel to the (001) surface. The unit cell data (lattice parameters and atomic coordinates) for *ε*_1_, *ε*_3_ and *ε*_4_ deformed OHAp are reported in Table 2, Table 3, Table 4, respectively. In Eq. (1), *δ* represents a multiplicative factor used to control the compressive (*δ* > 0) or tensile (*δ* < 0) deformation of the OHAp unit cell. Four unit cell configurations (two in expansion, *δ* = − 0.04 and *δ* = − 0.02, and two in compression, *δ* = + 0.02 and *δ* = + 0.04) were geometrically optimized for each considered strain (*ε*_*1*_, *ε*_*3*_, *ε*_*4*_), resulting in twelve deformed structures of OHAp. In the case of normal strain (*ε*_1_ and *ε*_3_), the unit cell was expanded/contracted by ± 4% and ± 2%, with resulting applied stress in the range ± 9 GPa. Symmetry analysis conducted on the deformed geometries revealed that for strain *ε*_1_ the OHAp unit cell belongs to space group *P*2_1_, for strain *ε*_3_ to *P*6_3_ and *ε*_4_ to space group *P*1 (absence of symmetry).

**Table 1:** Simulated OHAp lattice parameters (*a*, *b* and *c* reported in Å; *α*, *β* and *γ* in degrees) and internal coordinates of each irreducible atom (relative to s.g. *P*6_3_) at equilibrium.

*Equilibrium*					
*a*		9.38593	*α*		90
*b*		9.38593	*β*		90
*c*		6.87087	*γ*		120
Ca1	X	0.333333	O2	X	− 0.412019
	Y	− 0.333333		Y	0.465624
	Z	− 0.000114		Z	0.244387
					
Ca2	X	− 0.333333	O3	X	0.334650
	Y	0.333333		Y	0.251336
	Z	− 0.001445		Z	0.071593
					
Ca3	X	0.247056	O4	X	− 0.347179
	Y	− 0.004869		Y	− 0.256809
	Z	0.249398		Z	− 0.065903
					
P1	X	0.396585	O5	X	0.000000
	Y	0.366658		Y	0.000000
	Z	0.250675		Z	− 0.211631
					
O1	X	0.324502	H1	X	0.000000
	Y	0.483345		Y	0.000000
	Z	0.252703		Z	− 0.070371

**Table 2:** Simulated OHAp lattice parameters (*a*, *b* and *c* reported in Å; *α*, *β* and *γ* in degrees) and internal coordinates of each irreducible atom (relative to s.g. *P*2_1_) under the effect of strain *ε*_1_.

		*δ* = + 0.04	*δ* = + 0.02	*δ* = + 0.02	*δ* = + 0.04			*δ* = + 0.04	*δ* = + 0.02	*δ* = + 0.02	*δ* = + 0.04
*a*		9.10581	9.2455	9.52707	9.66888	*α*		90.00	90.00	90.00	90.00
*b*		9.38593	9.38593	9.38593	9.38593	*β*		90.00	90.00	90.00	90.00
*c*		6.87087	6.87087	6.87087	6.87087	*γ*		120.50	120.50	119.51	119.04
Ca1	X	0.334810	0.334094	0.332927	0.332281	O4	X	− 0.407217	− 0.409670	− 0.414299	− 0.416287
	Y	− 0.331032	− 0.332457	− 0.333257	− 0.333138		Y	0.464545	0.465670	0.464858	0.463486
	Z	− 0.002772	− 0.001538	0.000396	0.001281		Z	0.240659	0.242976	0.245895	0.247193
											
Ca2	X	− 0.335426	− 0.334529	− 0.332699	− 0.331698	O5	X	− 0.469999	− 0.467559	− 0.464408	− 0.463039
	Y	0.331427	0.332667	0.333309	0.333293		Y	0.117859	0.120143	0.123902	0.125989
	Z	− 0.002140	− 0.001585	− 0.001351	− 0.001216		Z	0.239863	0.242299	0.245715	0.247234
											
Ca3	X	0.238849	0.243491	0.249691	0.252164	O6	X	− 0.120787	− 0.121688	− 0.123114	− 0.123577
	Y	− 0.012941	− 0.008877	− 0.001104	0.002777		Y	0.410566	0.411251	0.412787	0.413645
	Z	0.247401	0.248632	0.249881	0.250231		Z	0.240694	0.242877	0.245898	0.247425
											
Ca4	X	0.012074	0.008724	0.000618	− 0.004550	O7	X	0.326445	0.331460	0.337021	0.338766
	Y	0.254700	0.253625	0.250064	0.247442		Y	0.246297	0.249132	0.253683	0.255873
	Z	0.248849	0.249276	0.249949	0.250410		Z	0.073655	0.072288	0.071044	0.070760
											
Ca5	X	− 0.256111	− 0.253600	− 0.250597	− 0.249725	O8	X	− 0.245517	− 0.248248	− 0.254588	− 0.258257
	Y	− 0.248862	− 0.247339	− 0.247559	− 0.248625		Y	0.085670	0.084471	0.082113	0.080969
	Z	0.249720	0.249546	0.249725	0.249876		Z	0.069842	0.070934	0.072006	0.072041
											
P1	X	0.395158	0.395861	0.397267	0.398207	O9	X	− 0.082900	− 0.083337	− 0.082818	− 0.082408
	Y	0.364127	0.365397	0.368077	0.369622		Y	− 0.333158	− 0.334300	− 0.334646	− 0.334680
	Z	0.250976	0.250771	0.250699	0.250741		Z	0.073071	0.072278	0.071116	0.070702
											
P2	X	− 0.364316	− 0.365317	− 0.368288	− 0.370176	O10	X	− 0.346732	− 0.347075	− 0.346556	− 0.345796
	Y	0.030767	0.030307	0.029306	0.028805		Y	− 0.255203	− 0.255916	− 0.257720	− 0.258815
	Z	0.248902	0.249928	0.251094	0.251384		Z	− 0.064935	− 0.065518	− 0.066304	− 0.066697
											
P3	X	− 0.029861	− 0.029834	− 0.029849	− 0.029788	O11	X	0.253804	0.255077	0.259281	0.261861
	Y	− 0.397445	− 0.396998	− 0.396220	− 0.395826		Y	− 0.095908	− 0.092927	− 0.087972	− 0.085534
	Z	0.250235	0.250525	0.250926	0.251265		Z	− 0.066095	− 0.065884	− 0.065922	− 0.066167
											
O1	X	0.324735	0.323687	0.326660	0.330157	O12	X	0.093661	0.091860	0.088316	0.086403
	Y	0.482800	0.482319	0.485632	0.488963		Y	0.352673	0.349651	0.344521	0.342029
	Z	0.254065	0.253318	0.252327	0.251940		Z	− 0.065452	− 0.065756	− 0.065818	− 0.065559
											
O2	X	− 0.479400	− 0.481514	− 0.484890	− 0.486650	O13	X	0.004396	0.001682	− 0.000994	− 0.001575
	Y	− 0.160573	− 0.159795	− 0.158046	− 0.156986		Y	0.000806	0.000320	− 0.000271	− 0.000473
	Z	0.252610	0.252714	0.252693	0.252461		Z	− 0.203916	− 0.208469	− 0.214420	− 0.217080
											
O3	X	0.166247	0.162538	0.155331	0.151899	H1	X	0.002633	0.000802	− 0.000841	− 0.001210
	Y	− 0.319183	− 0.321969	− 0.327414	− 0.330061		Y	− 0.000019	− 0.000395	0.000372	0.001256
	Z	0.252679	0.252709	0.252524	0.252451		Z	− 0.062913	− 0.067321	− 0.073074	− 0.075674

**Table 3:** Simulated OHAp lattice parameters (*a*, *b* and *c* reported in Å; *α*, *β* and *γ* in degrees) and internal coordinates of each irreducible atom (relative to s.g. *P*6_3_) under the effect of strain *ε*_3_.

		*δ* = + 0.04	*δ* = + 0.02	*δ* = + 0.02	*δ* = + 0.04			*δ* = + 0.04	*δ* = + 0.02	*δ* = + 0.02	*δ* = + 0.04
*a*		9.38593	9.38593	9.38593	9.38593	*α*		90.00	90.00	90.00	90.00
*b*		9.38593	9.38593	9.38593	9.38593	*β*		90.00	90.00	90.00	90.00
*c*		6.59603	6.73345	7.00828	7.1457	*γ*		120.00	120.00	120.00	120.00
Ca1	X	0.333333	0.333333	0.333333	0.333333	O2	X	− 0.409655	− 0.410986	− 0.412928	− 0.413543
	Y	− 0.333333	− 0.333333	− 0.333333	− 0.333333		Y	0.466622	0.465970	0.465637	0.465740
	Z	− 0.000031	− 0.000278	− 0.000904	− 0.001605		Z	0.244320	0.244309	0.243946	0.243187
											
Ca2	X	− 0.333333	− 0.333333	− 0.333333	− 0.333333	O3	X	0.337166	0.335544	0.333634	0.332652
	Y	0.333333	0.333333	0.333333	0.333333		Y	0.252233	0.251681	0.250917	0.250429
	Z	− 0.003014	− 0.002237	− 0.000716	− 0.000374		Z	0.066124	0.068984	0.074434	0.077408
											
Ca3	X	0.247451	0.247081	0.246898	0.246772	O4	X	− 0.348993	− 0.347836	− 0.347275	− 0.347891
	Y	0.000046	− 0.002840	− 0.006431	− 0.007450		Y	− 0.257121	− 0.256932	− 0.256962	− 0.257259
	Z	0.248920	0.249246	0.249385	0.249155		Z	− 0.061829	− 0.063824	− 0.067928	− 0.069991
											
P1	X	0.399551	0.397883	0.395473	0.394719	O5	X	0.000000	0.000000	0.000000	0.000000
	Y	0.369103	0.367792	0.365772	0.365044		Y	0.000000	0.000000	0.000000	0.000000
	Z	0.250104	0.250441	0.250902	0.251102		Z	− 0.208776	− 0.210548	− 0.211885	− 0.211451
											
O1	X	0.329540	0.326911	0.322026	0.319982	H1	X	0.000000	0.000000	0.000000	0.000000
	Y	0.486221	0.484797	0.481844	0.480488		Y	0.000000	0.000000	0.000000	0.000000
	Z	0.251860	0.252352	0.253216	0.253807		Z	− 0.061677	− 0.066456	− 0.073360	− 0.075575

**Table 4:** Simulated OHAp lattice parameters (*a*, *b* and *c* reported in Å; *α*, *β* and *γ* in degrees) and internal coordinates of each irreducible atom (relative to s.g. *P*1) under the effect of strain *ε*_4_.

		*δ* = + 0.04	*δ* = + 0.02	*δ* = + 0.02	*δ* = + 0.04			*δ* = + 0.04	*δ* = + 0.02	*δ* = + 0.02	*δ* = + 0.04
*a*		9.38781	9.38640	9.38640	9.38781	*α*		94.58	92.29	92.29	94.58
*b*		9.39344	9.38781	9.38781	9.39344	*β*		87.71	88.85	88.85	87.71
*c*		6.87636	6.87224	6.87224	6.87636	*γ*		120.02	120.01	120.01	120.02
Ca1	X	0.317777	0.325394	0.345306	0.350730	O7	X	− 0.424147	− 0.416872	− 0.414115	− 0.421470
	Y	− 0.335664	− 0.339535	− 0.318267	− 0.308686		Y	0.460737	0.464704	0.464319	0.459810
	Z	− 0.004382	− 0.000749	0.000061	0.003185		Z	0.220911	0.236204	0.253665	0.272171
											
Ca2	X	− 0.350739	− 0.345318	− 0.325344	− 0.317811	O8	X	0.421491	0.414110	0.416883	0.424165
	Y	0.308678	0.318245	0.339541	0.335575		Y	− 0.459857	− 0.464346	− 0.464694	− 0.460757
	Z	− 0.496843	− 0.499870	0.499292	0.495590		Z	− 0.227839	− 0.246542	− 0.263921	− 0.279192
											
Ca3	X	− 0.316706	− 0.322659	− 0.347882	− 0.353545	O9	X	− 0.471770	− 0.467099	− 0.471200	− 0.475702
	Y	0.339178	0.343603	0.315378	0.307537		Y	0.114190	0.120651	0.116920	0.111324
	Z	0.003451	− 0.000631	− 0.000976	− 0.003025		Z	0.225423	0.226771	0.263951	0.268135
											
Ca4	X	0.353566	0.347874	0.322603	0.316635	O10	X	− 0.121780	− 0.122116	− 0.122702	− 0.122305
	Y	− 0.307562	− 0.315382	− 0.343604	− 0.339244		Y	0.416154	0.414081	0.416816	0.419510
	Z	0.497034	0.498979	0.499305	− 0.496550		Z	0.280222	0.268411	0.221001	0.212080
											
Ca5	X	0.238172	0.244317	0.240684	0.235374	O11	X	0.122281	0.122688	0.122093	0.121789
	Y	− 0.007066	− 0.005546	− 0.007633	− 0.008280		Y	− 0.419475	− 0.416855	− 0.414034	− 0.416116
	Z	0.263151	0.255414	0.243789	0.236597		Z	− 0.287908	− 0.279002	− 0.231674	− 0.219790
											
Ca6	X	− 0.235385	− 0.240627	− 0.244328	− 0.238180	O12	X	0.475653	0.471260	0.467054	0.471780
	Y	0.008264	0.007666	0.005526	0.007063		Y	− 0.111372	− 0.116880	− 0.120669	− 0.114178
	Z	− 0.263314	− 0.256208	− 0.244574	− 0.236824		Z	− 0.231836	− 0.236129	− 0.273243	− 0.274632
											
Ca7	X	0.011899	0.008172	0.004476	0.007596	O13	X	0.294097	0.314600	0.348475	0.357871
	Y	0.255380	0.254440	0.255109	0.256278		Y	0.232824	0.243480	0.256793	0.264575
	Z	0.245777	0.250077	0.248823	0.252624		Z	0.092206	0.083082	0.062531	0.058012
											
Ca8	X	− 0.255123	− 0.252422	− 0.253419	− 0.257677	O14	X	− 0.357847	− 0.348406	− 0.314463	− 0.293989
	Y	− 0.251298	− 0.247303	− 0.247645	− 0.252167		Y	− 0.264600	− 0.256753	− 0.243430	− 0.232812
	Z	0.238718	0.243382	0.256264	0.261076		Z	− 0.441962	− 0.437439	− 0.416837	− 0.407743
											
Ca9	X	0.257719	0.253400	0.252409	0.255165	O15	X	− 0.239997	− 0.241474	− 0.265029	− 0.269416
	Y	0.252215	0.247583	0.247319	0.251278		Y	0.089710	0.085247	0.085724	0.085911
	Z	− 0.238915	− 0.243751	− 0.256653	− 0.261272		Z	0.067441	0.071672	0.071015	0.074323
											
Ca10	X	− 0.007549	− 0.004481	− 0.008189	− 0.011967	O16	X	− 0.091499	− 0.090552	− 0.070627	− 0.068819
	Y	− 0.256235	− 0.255130	− 0.254376	− 0.255382		Y	− 0.367644	− 0.357511	− 0.305906	− 0.295881
	Z	− 0.247446	− 0.251170	− 0.249952	− 0.254167		Z	0.065286	0.064135	0.081394	0.080736
											
P1	X	0.387784	0.392693	0.394568	0.389046	O17	X	0.068879	0.070548	0.090518	0.091524
	Y	0.363307	0.365549	0.365743	0.363954		Y	0.295981	0.305844	0.357462	0.367689
	Z	0.259845	0.257975	0.243578	0.241845		Z	− 0.419296	− 0.418581	− 0.435836	− 0.434678
											
P2	X	− 0.389017	− 0.394562	− 0.392695	− 0.387777	O18	X	0.269374	0.265004	0.241449	0.239960
	Y	− 0.363976	− 0.365739	− 0.365571	− 0.363317		Y	− 0.085944	− 0.085783	− 0.085235	− 0.089713
	Z	− 0.258135	− 0.256412	− 0.242030	− 0.240183		Z	− 0.425671	− 0.428976	− 0.428328	− 0.432568
											
P3	X	− 0.367292	− 0.366735	− 0.368260	− 0.368541	O19	X	− 0.302262	− 0.328259	− 0.357389	− 0.364360
	Y	0.028048	0.030259	0.028740	0.027573		Y	− 0.236288	− 0.248801	− 0.261053	− 0.266613
	Z	0.235070	0.241021	0.260623	0.267007		Z	− 0.086547	− 0.075973	− 0.058820	− 0.056118
											
P4	X	− 0.028723	− 0.028859	− 0.028823	− 0.028992	O20	X	0.364312	0.357338	0.328103	0.302313
	Y	− 0.394558	− 0.395317	− 0.393824	− 0.392640		Y	0.266572	0.260987	0.248735	0.236280
	Z	0.257796	0.253098	0.247908	0.243674		Z	0.443890	0.441155	0.423940	0.413440
											
P5	X	0.028992	0.028805	0.028865	0.028743	O21	X	0.245316	0.247494	0.269866	0.274226
	Y	0.392671	0.393793	0.395355	0.394586		Y	− 0.095802	− 0.092378	− 0.090265	− 0.087929
	Z	− 0.256317	− 0.252053	− 0.246906	− 0.242159		Z	− 0.062120	− 0.065850	− 0.065471	− 0.068438
											
P6	X	0.368504	0.368268	0.366731	0.367270	O22	X	0.094696	0.094350	0.079329	0.075152
	Y	− 0.027606	− 0.028760	− 0.030237	− 0.028078		Y	0.373387	0.365363	0.320241	0.307422
	Z	− 0.232990	− 0.239389	− 0.258990	− 0.264961		Z	− 0.061900	− 0.060497	− 0.074635	− 0.074407
											
O1	X	0.316110	0.320362	0.322261	0.318653	O23	X	− 0.075131	− 0.079222	− 0.094332	− 0.094689
	Y	0.480524	0.482163	0.482437	0.482438		Y	− 0.307374	− 0.320080	− 0.365381	− 0.373399
	Z	0.280226	0.270527	0.235184	0.225712		Z	0.425550	0.425321	0.439479	0.438105
											
O2	X	− 0.318623	− 0.322250	− 0.320379	− 0.316106	O24	X	− 0.274193	− 0.269836	− 0.247486	− 0.245369
	Y	− 0.482462	− 0.482432	− 0.482195	− 0.480534		Y	0.087944	0.090310	0.092335	0.095788
	Z	− 0.274270	− 0.264797	− 0.229445	− 0.219720		Z	0.431558	0.434516	0.434159	0.437889
											
O3	X	− 0.477362	− 0.481229	− 0.479654	− 0.476029	O25	X	0.012018	0.004040	0.002550	0.009398
	Y	− 0.162376	− 0.159121	− 0.160975	− 0.162981		Y	0.004631	− 0.000335	− 0.002154	− 0.000222
	Z	0.226760	0.238412	0.266527	0.277438		Z	− 0.218148	− 0.213933	− 0.210326	− 0.209714
											
O4	X	0.159946	0.159757	0.159432	0.159360	O26	X	− 0.009426	− 0.002549	− 0.004045	− 0.012063
	Y	− 0.321765	− 0.323181	− 0.324593	− 0.322395		Y	0.000251	0.002210	0.000317	− 0.004566
	Z	0.251920	0.248974	0.254990	0.251836		Z	0.290258	0.289557	0.285960	0.281846
											
O5	X	− 0.159362	− 0.159447	− 0.159762	− 0.159926	H1	X	0.000821	− 0.000434	0.004726	0.011511
	Y	0.322395	0.324573	0.323147	0.321769		Y	0.001723	− 0.003302	0.001300	− 0.001104
	Z	− 0.248142	− 0.244909	− 0.250952	− 0.248073		Z	− 0.077412	− 0.072863	− 0.069308	− 0.068463
											
O6	X	0.476043	0.479636	0.481252	0.477346	H2	X	− 0.011516	− 0.004702	0.000488	− 0.000875
	Y	0.162940	0.160961	0.159139	0.162351		Y	0.001220	− 0.001243	0.003345	− 0.001712
	Z	− 0.222613	− 0.233535	− 0.261582	− 0.273231		Z	0.431514	0.430576	0.427022	0.422587

### 1.2 Vibrational frequencies

For each optimized hydroxylapatite model, both at equilibrium (*δ* = + 0.00) and at strained configurations (*δ* = ± 0.02 and *δ* = ± 0.04), vibrational frequencies (Raman and IR) were obtained by means of finite displacements method. In the OHAp unit cell, there are 44 atoms, resulting in 132 degrees of freedom (129 with vibrational character) [1]. The calculated frequencies for *ε*_1_, *ε*_3_ and *ε*_4_ deformed OHAp are reported in Tables S1–S3 (Supplementary material), respectively. To aid the comparison between the peak positions, for the strains *ε*_1_ and *ε*_4_ the normal modes of the non-deformed hydroxylapatite were calculated with the symmetry of the strained cells (*P*2_1_ and *P*1, respectively).

### 1.3 Vibrational intensities

Vibrational intensities were calculated within the Placzek approximation (Raman, partial derivatives of the polarizability tensor with respect to atomic positions) [3], [4], [5] and analytically through Coupled-Perturbed Kohn–Sham approach (Infrared) [6], [7], [8], [9]. The intensity of each normal mode, IR and Raman, was calculated for each OHAp model and reported in the Supplementary material section (Tables S1–S3).

## 2 Theoretical design, materials, and methods

The data here presented was obtained by first principle simulations on periodic systems, using the CRYSTAL14 code [10], which implements the Hartree–Fock and Kohn–Sham self-consistent field method.

### 2.1 Basis set

Multielectron wave functions are constructed as an antisymmetrized product (Slater determinant) of monoelectronic crystalline orbitals (CO) that are linear combination of local functions (atomic orbitals, AO) centred on each atom in the system. In turn, atomic orbitals (basis set) are linear combinations of Gaussian-type functions (GTF). The all-electron basis sets employed in the present simulations were chosen among previously adopted ones for [2], [11], [12], [13].

### 2.2 Hamiltonian and computational parameters

The Becke [14] three-parameter (B3LYP) hybrid exchange functional in combination with the gradient-corrected correlation functional of Lee et al. [15] has been adopted for all calculations. The exchange–correlation contribution is performed over a grid of points and is the result of a numerical integration of the electron density and its gradient. The adopted pruned grid is given by 75 points and 974 angular points (XLGRID) and obtained from The Gauss–Legendre quadrature and Lebedev schemes [16]. The tolerance thresholds that control accuracy of the Coulomb and exchange integrals were set to 10^−7^ and 10^−16^, respectively [10]. The Hamiltonian matrix has been diagonalized using a shrinking factor that leads to 12 reciprocal lattice points (k-points). The convergence on total energy was reached when the difference between the energy of two subsequent self-consistent field cycles was less than 10^−8^ Hartree.

Van der Waals (dispersive) forces were included with the (DFT+D2 scheme [17], which adds the following contribution to the calculated DFT energy:

(2) $$E_{DISP}=-s_{6}\sum_{g}\sum_{i\neq j}f_{dump}\left(R_{ij,g}^{6}\right)\frac{C_{6}^{i}C_{6}^{j}}{R_{ij,g}^{6}}$$

The summation over all atom pairs *ij* and **g** lattice vectors excludes the self- interaction contribution (*i = j*) for every **g**. The parameters *C*_*6*_^*i*^ represent the dispersion coefficient for the atom *i*, *R*_*ij,***g**_ is the interatomic distance between atom *i* in the reference cell and atom *j* in the neighbouring cells at distance |**g**| and *s*_*6*_ is a functional-dependent scaling factor. The function *f*_*dump*_ is used to dump the energy correction to avoid double counting of short-range contributions to the energy and depends on the sum of atomic van der Waals radii and on a steepness parameter (*d* = 20). Due to the molecular nature of the DFT+D2 scheme, which tends to overestimate cohesive energy in solid crystals, the original B3LYP+D parameters where modified, setting *s*_*6*_ to 1, *R*_*vdw*_(H) to 1.30 and the heavier atom van der Waals radii were scaled by a factor 1.05 (B3LYP-D* approach) [18], [19], [20], [21], [22], [23].

### 2.3 Vibrational calculations

In periodic systems and within the harmonic approximation, the phonon frequencies at Γ point are evaluated diagonalising the central zone (*k* = 0) mass-weighted Hessian matrix:

(3) $$W_{ij}\left(k=0\right)=\sum_{G}\frac{H_{ij}^{0G}}{\sqrt{M_{i}M_{j}}}$$

$H_{ij}^{0G}$ is the second derivative of the electronic and nuclear repulsion energy E evaluated at equilibrium **u** ***=*** **0** with respect to the displacement of atom A in cell 0 ($u_{i}=x_{i}-x_{i}^{*}$) and displacement of atom B in cell G ($u_{j}=x_{j}-x_{j}^{*}$) from their equilibrium position $x_{i}^{*}$, $x_{j}^{*}$:

(4) $$\sum_{G}H_{ij}^{0G}=\sum_{G}\begin{array}{c} {\left[\frac{\partial^{2}E}{\partial u_{i}^{0}\partial u_{j}^{G}}\right]}_{0}\\ i=1,\ldots ,3N;\;j=1,\ldots ,3N \end{array}$$

In CRYSTAL14, the calculation of the Hessian at equilibrium is made by the analytical evaluation of the energy first derivatives, $\Phi_{j}$ of *E* with respect to the atomic displacements:

(5) $$\Phi_{j}=\sum_{G}\nu_{j}^{G}=\sum_{G}\frac{\partial E}{\partial u_{j}^{G}}\;j=1,\ldots ,3N$$

while second derivatives at **u = 0** (where all first derivatives are zero) are calculated numerically using a "two-point" formula:

(6) $$\begin{array}{c} {\left[\frac{\partial \Phi_{j}}{\partial u_{i}^{0}}\right]}_{0}\approx \frac{\Phi_{j}\left(0,\ldots ,u_{i}^{0},\ldots ,0\right)-\Phi_{j}\left(0,\ldots ,u_{i}^{0},\ldots ,0\right)}{u_{i}^{0}}\\ i=1,\ldots ,3N;\;j=1,\ldots ,3N \end{array}$$

The Hessian matrix eigenvalues provide the normal harmonic frequencies *ω*_*h*_ and it is obtained with 3*N* + 1 SCF and gradient calculation.
